# Operator decision-making in angiography-only guided revascularization for lesions not indicated for FFR: a QFR-based functional assessment in chronic coronary syndrome

**DOI:** 10.3389/fcvm.2024.1336341

**Published:** 2024-02-26

**Authors:** Sadeek S. Kanoun Schnur, Robertas Pranevičius, Cosima Stark, Dan Prunea, Judit Andreka, Albrecht Schmidt, Stefan Harb, Zoltan Ruzsa, Robert Zweiker, Jesse Kane, Gabor G. Toth

**Affiliations:** ^1^ Department of Cardiology, University Heart Center Graz, Medical University Graz, Graz, Austria; ^2^ Peninsula Deanery, Royal Devon University Healthcare NHS Foundation Trust, Exeter, United Kingdom; ^3^ Doctoral School of Clinical Medicine, Faculty of Medicine, University of Szeged, Szeged, Hungary; ^4^ Department of Cardiology, Heart and Vascular Center, Republican Siauliai Hospital, Siauliai, Lithuania; ^5^ “Niculae Stancioiu” Heart Institute, University of Medicine “Iuliu Hatieganu”, Cluj-Napoca, Romania; ^6^ Department of Cardiology, University of Vermont Larner College of Medicine, Burlington, VT, United States

**Keywords:** chronic coronary syndrome, functional misclassification, fractional flow reserve, quantitative flow ratio, angiography guided PCI, QFR, FFR

## Abstract

**Background:**

Discordance between coronary angiographic findings and invasive functional significance is well-established. Yet, the prevalence of this mismatch in an era increasingly utilizing invasive functional assessments, such as fractional flow reserve (FFR), remains unclear. This study examines the extent of such discrepancies in current clinical practice.

**Methods:**

This single-center prospective registry included consecutive patients with chronic coronary syndrome (CCS) who underwent elective coronary angiography, with or without revascularization. Coronary angiograms deemed not requiring FFR due to clear anatomical distinctions, either anatomically severe indicating a need for revascularization or mild suggesting no need for intervention, were selected for evaluation. These were then subjected to post-hoc analysis by three independent operators who were blinded to the definitive treatment strategies. Importantly, the post-hoc analysis was conducted in two distinct phases: firstly, a re-evaluation of coronary stenosis, and secondly, a separate functional assessment, each carried out independently. Coronary stenosis severity was assessed visually, while functional relevance was determined by quantitative flow ratio (QFR), calculated using a computational fluid dynamics algorithm applied to angiographic images. Analysis focused on discrepancies between QFR-based functional indications and revascularization strategies actually performed.

**Results:**

In 191 patients, 488 vessels were analyzed. Average diameter stenosis (DS) was 37 ± 34%, and QFR was 0.87 ± 0.15, demonstrating a moderate correlation (*r* = −0.84; 95% CI: −0.86 to −0.81, *p* < 0.01). Agreement with QFR at conventional anatomical cutoffs was 88% for 50% DS and 91% for 70% DS. Mismatches between revascularization decisions and QFR indications occurred in 10% of cases. Discrepancies were more frequent in the left anterior descending artery (14%) compared to the left circumflex (6%) and the right coronary artery (9%; *p* = 0.07).

**Conclusion:**

In a cardiac-center where FFR utilization is high, discordance between coronary angiography and functional significance persists, even when operators are confident in their decisions *not* to use functional interrogation. This gap, most evident in the left anterior descending artery, highlights the potential need for integrated angiography-based functional assessments to refine revascularization decisions in CCS.

## Introduction

While coronary artery disease is the leading cause of death and morbidity worldwide, there has been much debate regarding the role of revascularization in Chronic Coronary Syndrome (CCS) (1, 2). However, numerous studies have corroborated the notion that the benefits of revascularization can only be fully realized when targeted towards coronary lesions responsible for significant reversible ischemia (3). Overtreatment by unnecessary revascularization or undertreatment with failure to perform intervention to a significant lesion are associated with short- and long-term hazard (4–7). Considering the limitations of coronary angiography (CAG) in detecting the functionally relevant coronary stenoses (8), functionally guided revascularization strategies have demonstrated its superiority compared to pure angiography-based decisions (4–7, 9–13). Still, the adoption of functional guidance has remained limited and shows marked variation between countries, centres and operators (14). This could predominantly be due to financial costs, but also to the practical burden associated with the procedure. The introduction of adenosine-free methods has, to some extent, brought a degree of simplification to the procedure with the aim of broader adoption. Yet, despite this, the rate of functionally-guided revascularizations is still around 15%–20% of all cases even in centres with highest usage rates (15).

Recently, non-wire-based functional assessments, which have been widely validated, have been introduced into clinical practice from CAG. These allow for both *ad hoc* and *post hoc* evaluations of the functional significance of coronary stenoses, based on three-dimensional angiogram-based estimates.

In this study, our aim was to explore the correlation between revascularization decisions derived from coronary angiography alone and actual functional significance, especially in a setting with high FFR use and for lesions not meeting the estimated anatomical thresholds for FFR.

## Methods

### Patient selection

**Figure 1:**
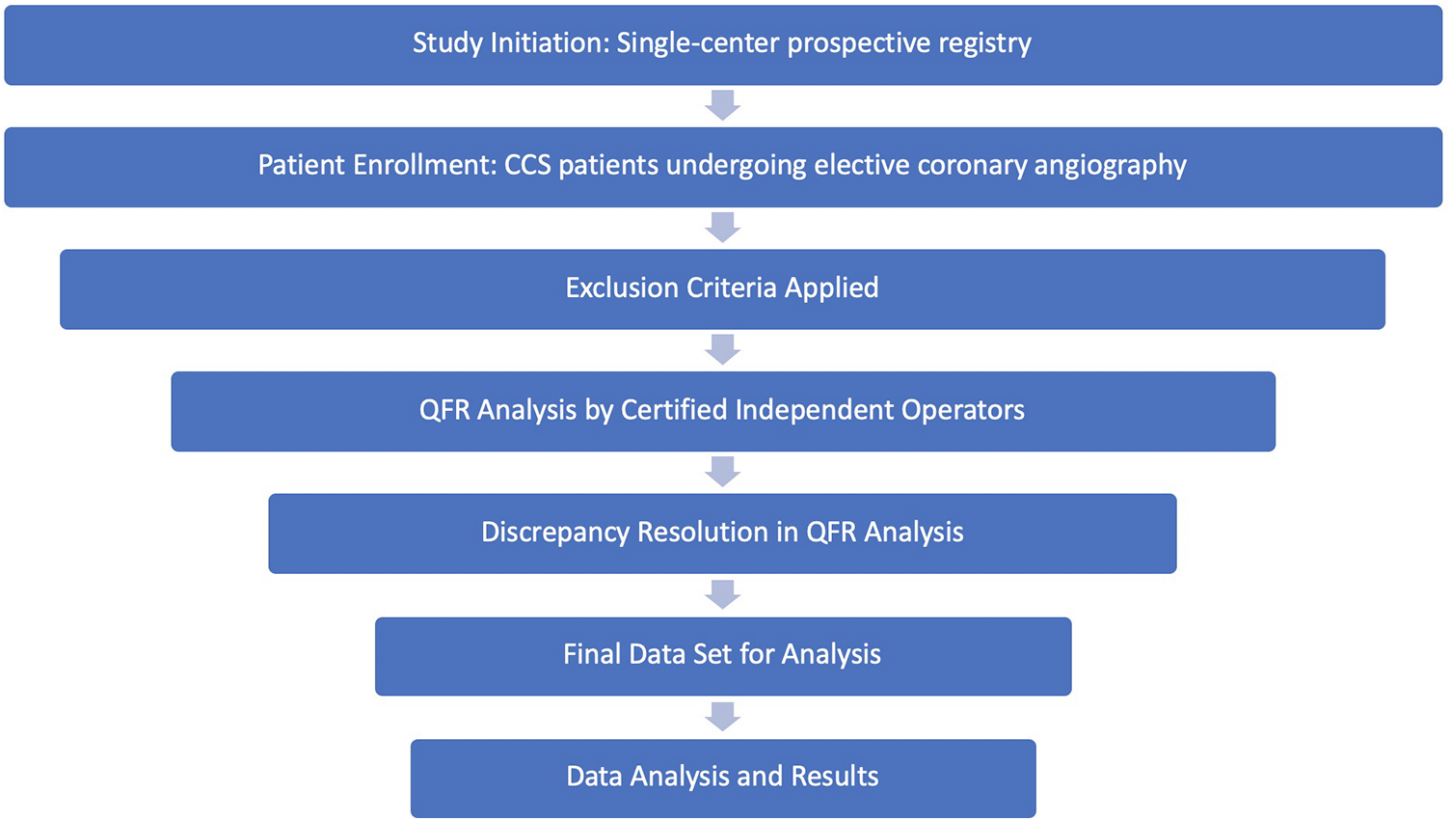
Study methodology flow chart. This flow chart illustrates the sequential steps of the study.

This single-center prospective registry included consecutive patients recruited over two months, who underwent elective coronary catheterization for CCS, with or without subsequent revascularization. Patients were excluded, if invasive physiologic- or intravascular imaging assessments were indicated. This indication was based on established parameters as dictated by current guidelines on the use of invasive functional assessments, such as FFR, to further evaluate lesions and their functional significance. It is important to note that the interpretation of these anatomical cutoffs was subject to the operator's own discretion as would be in every day clinical practice. Exclusion criteria also included previous CABG, significant valvular disease, and cases where revascularization was indicated but ultimately deferred based on overall clinical indications. Additionally, exclusions of quantitative flow ratio (QFR) assessments were incorporated, namely aorto-ostial lesions, chronic total occlusions, lack of appropriate projections for analysis, poor contrast opacification and atrial fibrillation Figure 1. The study was approved by the local ethics commission.

### Coronary angiography

Coronary angiography was performed according to standard of care, utilizing 6F diagnostic coronary catheters and multiple standard projections in order to visualize all coronary arteries in at least two orthogonal views. Quantitative Coronary Angiography was not performed for any of the cases. Indication and performance of revascularization was left to the operator's discretion, as well as the use of additional lesion assessment tools, such as invasive physiology or intravascular imaging. Patients, in whom invasive physiologic assessment was used, were excluded from the analysis.

### Coronary angiography assessment

Analysis was performed by three independent operators. They provided visually estimated diameter stenosis severity for all the three major coronary arteries (DS), this ranged from 0%–99%. Note, assessment was done prior to QFR analysis and therefore the analyzers were not aware of functional significance at this point. Additionally, the analyzers were blinded to the definitive revascularization decisions. In the event of a relevant discrepancy, the case was reviewed to meet consensus.

### Quantitative flow ratio

QFR, available at the Medis QAngio XA 3D and at the Pulse AngioPlus solution, was the first angiogram-based functional assessment tool, which demonstrated superiority to three-dimensional quantitative coronary angiography in the prediction of FFR values with 88% specificity and 84% sensitivity (16–18). It calculates FFR using three-dimensional reconstructions from two CAG projections separated by a minimum of 25 degrees.

QFR analysis was carried out *post hoc* on all three coronary arteries from the offline baseline angiogram using Medis QFR® software, developed by Medis Medical Imaging Systems. The analysis was completed by three independent operators certified in QFR analysis. The process involved a systematic nine-step protocol starting with the selection of appropriate angiographic frames, followed by delineation of vessel contours, and culminating in the computation of the FFR value utilizing the software's integrated algorithms. These operators were blinded to the definitive revascularization decisions, and any discrepancies were resolved through a consensus review process to ensure the accuracy and reliability of the QFR measurements.

### Analysis of revascularization strategies

The analysis focused on the discrepancy between QFR-based indications for revascularization and the definitive revascularization strategy. Definitive revascularization strategies were determined on two levels: the visual assessment of coronary stenosis (vessel-level), and the actual revascularization decision made for each individual patient (patient-level).

At the vessel level, treatment decisions were matched with the offline QFR assessment results and classified as (1) *Appropriate Revascularization*, when QFR suggested significant stenosis and revascularization was performed; (2) *Appropriate Deferral*, when QFR suggested no significant stenosis and no revascularization was performed; (3) *Inappropriate Revascularization*, when QFR suggested no significant stenosis but revascularization was performed; and (4) *Inappropriate Deferral*, when QFR suggested significant stenosis but no revascularization was performed.

At the patient level, the overall revascularization strategies were analyzed by matching the actual clinical revascularization decisions with the offline QFR assessment results. These strategies were classified as (a) *Overall Appropriate Revascularization Strategy*, where the decisions for each of the three coronary vessels aligned with the QFR assessment; (b*) Incomplete Revascularization*, where some lesions that warranted treatment based on QFR were not revascularized; and (c) *Functional Overtreatment*, where revascularization exceeded the recommendations suggested by QFR.

### Statistical methods

All analyses were performed with Prism GraphPad 9.0 (GraphPad Software Inc., California, US). Summary descriptive statistics are reported as mean ± SD or *n* (%), as appropriate. Normal distribution was tested by D'Agostino-Pearson omnibus normality test. Continuous variables were compared by Mann-Whitney tests or Kruskal-Wallis test and categorical variables were compared with Fisher's exact or chi-square tests, as appropriate. Sensitivity, specificity, diagnostic accuracy were described. Correlation was described by Pearson correlation coefficient. A probability value of *p* < 0.05 was considered as significant.

## Results

A total of 191 consecutive CCS patients were enrolled, with (*n* = 98) receiving revascularization and (*n* = 93) without revascularization. 68.9% of them were males and the average age was 72.3 years. Detailed patient characteristics are shown in Table 1. The analysis included a total of 488 vessels, with 37% being left anterior descending (LAD) vessels, 30% left circumflex (LCx) vessels, and 33% right coronary artery (RCA) vessels. The mean DS was 37% with a standard deviation of ±34%, the median DS was 30%, and the mean QFR was 0.87 ± 0.15.

**Table 1:** Clinical and coronary angiographic characteristics.

Characteristics	*N*	(%)
Gender		
Male	132	68.9%
Female	59	31.1%
NYHA classification		
Class 1	139	72.6%
Class 2	41	21.7%
Class 3	11	5.7%
Class 4	0	0.0%
CCS grading		
Grade 0	110	57.5%
Grade 1	27	14.1%
Grade 2	49	25.7%
Grade 3	5	1.9%
Grade 4	0	0.0%
Hypertension	146	76.4%
Hyperlipidemia	128	67.0%
Diabetes Mellitus	43	22.6%
Previous PCI	40	20.7%
Coronary angiographic characteristics		
SVD	57	30%
MVD	63	33%
LAD > 50% stenosis	83	43.6%
LAD > 70% stenosis	66	34.6%
LCx > 50% stenosis	25	13.3%
LCx > 70% stenosis	15	8.0%
RCA > 50% stenosis	29	15.4%
RCA > 70% stenosis	20	10.6%
Revascularization performed	87	45.7%

SVD, single vessel disease; MVD, multivessel disease (≥2 coronary vessel disease); LAD, left anterior descending; LCx, left circumflex; RCA, right coronary artery; PCI, percutaneous coronary intervention; NYHA, New York heart association; CCS, Canadian cardiovascular society.

**Figure 2:**
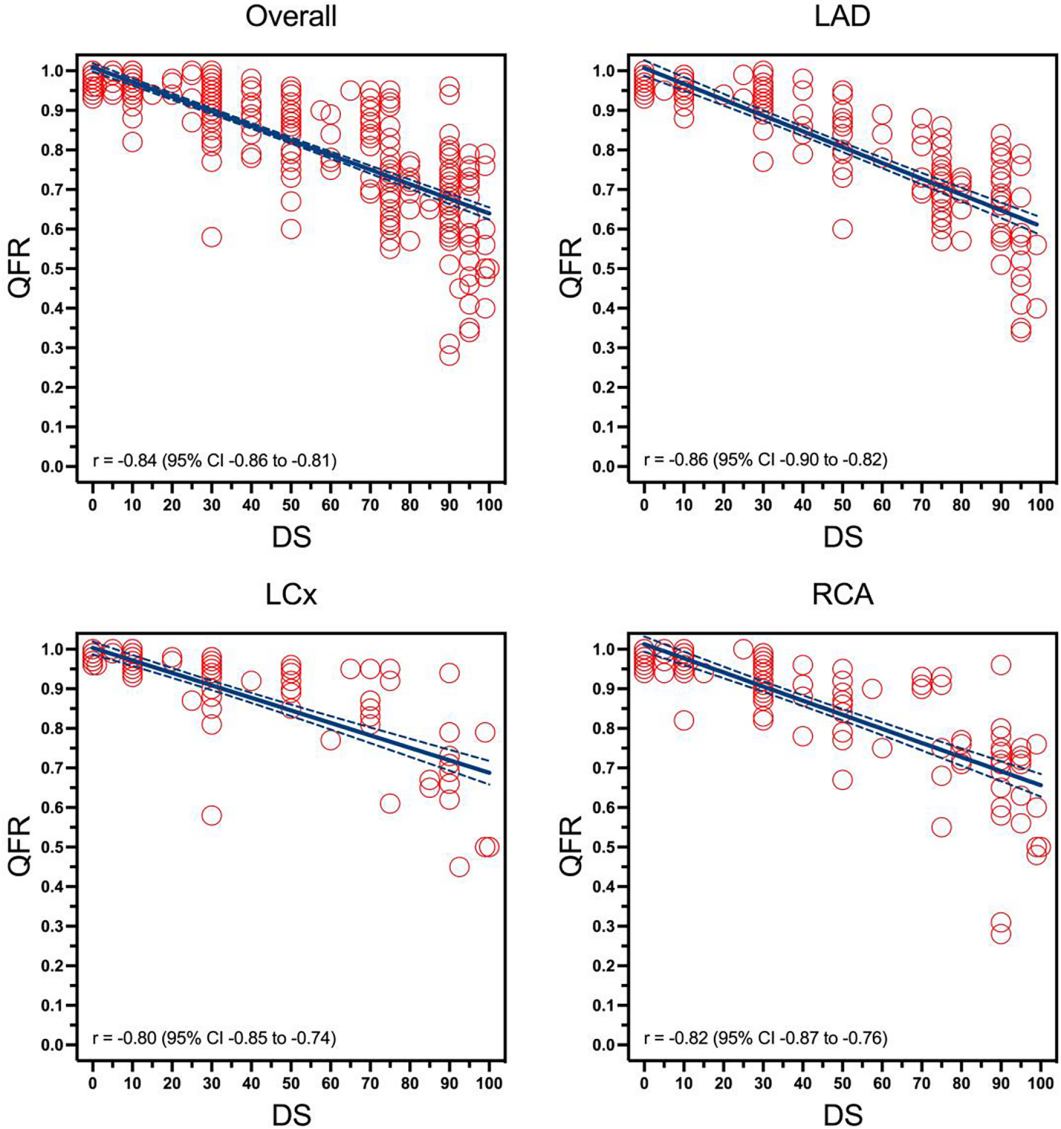
Correlation between visually estimated diameter stenosis and quantitative flow ratio overall in different coronary arteries. The figure illustrates four regression curves representing the relationship between angiographic severity (DS), represented by the X-axis, and functional significance determined by QFR analysis, represented by the Y-axis, in each of the coronary arteries (LAD, LCx, RCA) as well as an aggregate of all four arteries. OVERALL (an aggregate of all 3 coronary arteries), LAD, left anterior descending artery; LCX, left circumflex artery; RCA, right coronary artery; DS, diameter stenosis; QFR, quantitive flow ratio.

Overall, a moderate correlation was observed between angiographic severity (DS) and functional significance determined by QFR analysis (*r* = −0.84; 95% CI −0.86 to −0.81, *p* < 0.01) Figure 2. Correlation was found to be the strongest in the LAD (*r* = −0.86; 95% CI −0.90 to −0.82, *p* < 0.01), while weaker in the RCA (*r* = −0.82; 95% CI −0.87 to −0.76, *p* < 0.01) and the LCx (*r* = −0.80; 95% CI −0.85 to −0.74, *p* < 0.01).

When comparing the QFR-based functional significance with an arbitrary angiographic cut-off value of 50% DS, the overall agreement with QFR as the reference standard was 88%. In this analysis, QFR was used as the benchmark to evaluate the specificity and sensitivity of the angiographic assessment. The specificity of using a 50% DS cut-off for identifying lesions as significant compared to QFR was 84%, and the sensitivity was 97%. By taking the arbitrary angiographic cut-off value up to 70% DS, the overall agreement improves to 91% with specificity at 93% but a reduced sensitivity of 88%. Diagnostic accuracy of 50% DS cut-off (87%, 86%, and 90%, respectively) and of 70% DS cut-off values (90%, 92%, and 92%, respectively) were comparable in LAD, LCx and RCA Table 2.

**Table 2:** Diagnostic accuracy at two angiographic cut-off values according to coronary vessel.

50% DS cutoff						70% DS cutoff					
Agree	Disagree	SENS	SPEC	PPV	NPV	Agree	Disagree	SENS	SPEC	PPV	NPV
Overall											
87.50	12.50	96.88	84.17	68.51	98.70	91.39	8.61	87.50	92.78	81.16	95.43
LAD											
86.81	13.19	97.14	80.36	75.56	97.83	90.11	9.89	87.14	91.96	87.14	91.96
LCX											
85.81	14.19	95.00	84.38	48.72	99.08	91.89	8.11	90.00	92.19	64.29	98.33
RCA											
89.87	10.13	97.37	87.50	71.15	99.06	92.41	7.59	86.84	94.17	82.50	95.76

This table displays analytic metrics of angiography-driven revascularization decisions for different coronary arteries at 50% and 70% cut-off levels. The arteries evaluated are OVERALL (an aggregate of all coronary arteries), LAD, left anterior descending artery; LCX, left circumflex artery); RCA, right coronary artery.

Each column in the table represents a different analytic metric: “Agree”: percentage of cases where angiography-based decisions and functional assessments agreed, “Disagree”: percentage of cases where angiography-based decisions disagreed with functional assessments sensitivity (SENS), specificity (SPEC), positive predictive value (PPV), and negative predictive value (NPV).

**Figure 3:**
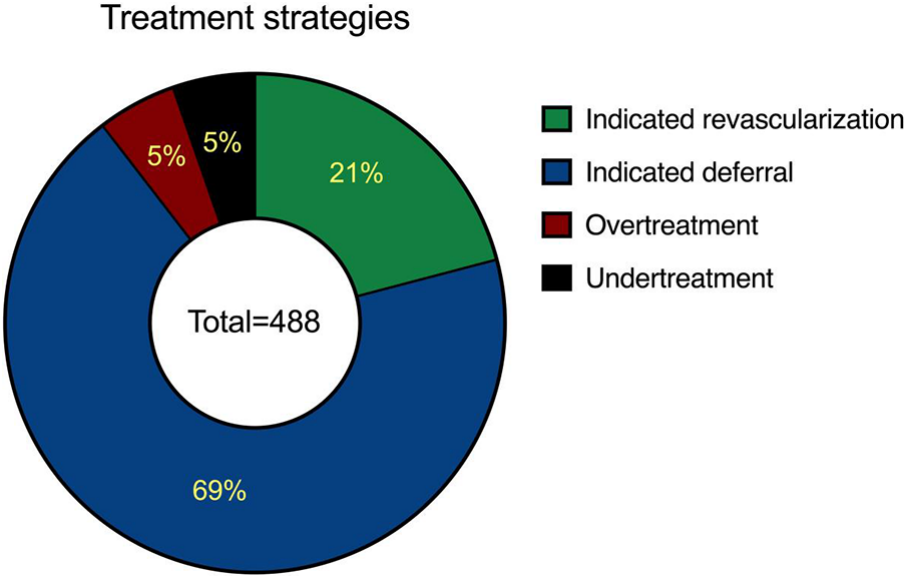
Distribution of revascularization strategies over the entire cohort. Pie chart illustrating the relationship between actual revascularization decisions and QFR indications across 488 analyzed vessels. It shows that 90% of revascularization decisions were in agreement with QFR-based indications, comprising 21% *appropriate revascularizations* and 69% *appropriate deferrals*, both indicating concordance. The remaining 10% of decisions were discordant with the functional indication, including 5% *inappropriate revascularizations* and 5% *inappropriate deferrals*, reflecting a lack of concordance.

In the investigated cases, operators' angiogram-based decision led to revascularization in a total of 127 vessels (26%), while the remaining vessels were deemed suitable for conservative treatment. In 437 vessels (90%), revascularization decisions showed agreement with the QFR-based indication. Among these 21% were considered *appropriate revascularizations* and 69% *appropriate deferrals.* Contrarily, in 51 vessels (10%), revascularization decisions were discrepant with the functional indication. This included 5.0% cases of *inappropriate revascularization*s and 5.0% cases of *inappropriate deferral*s Figure 3.

**Figure 4:**
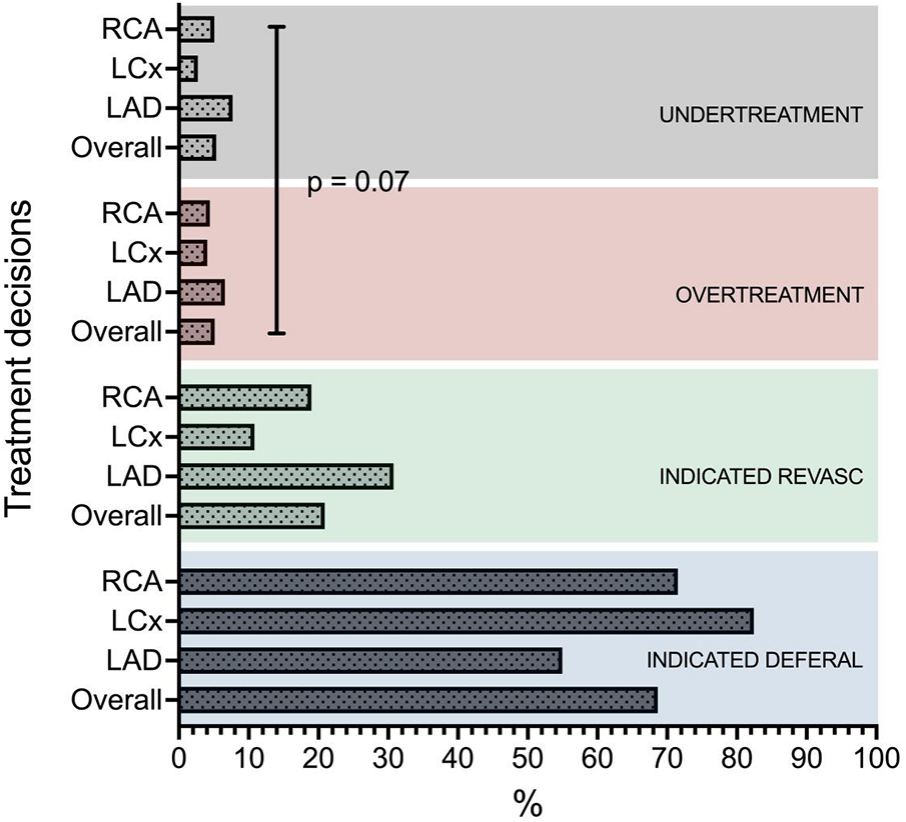
Distribution of revascularization strategies in the different coronary arteries. OVERALL (an aggregate of all 3 coronary arteries), LAD, left anterior descending artery; LCX, left circumflex artery; RCA, right coronary artery.

For vessels with *inappropriate deferral,* QFR was 0.67 ± 0.13, with no significant difference compared to QFR for *appropriate revascularization* (0.65 ± 0.12; *p* = 0.18). Meanwhile, QFR for vessels with *inappropriate revascularization* was 0.88 ± 0.05, markedly lower than for vessels with *appropriate deferrals* (0.95 ± 0.04; *p* = 0 < 0.01). Strategic discrepancy was most frequently observed in the LAD with 14.3% inappropriate decisions (7.7% *inappropriate deferrals* and 6.6% *inappropriate revascularizations* of all decisions), which is notably higher than in the LCx (6.8%) and RCA (9.5%) (*p* = 0.07) Figure 4.

Three-vessel QFR was available in 160 patients (84%). Here an overall appropriate revascularization strategy was observed in 75% of cases. 21 patients (13%) were left with incomplete revascularization, while 21 patients (13%), there was functional overtreatment. Both over- and undertreatment were noted in 1% of patients.

## Discussion

The study aimed to evaluate the correlation between revascularization decisions based solely on coronary angiography and the physiological significance as determined by QFR in patients with chronic coronary syndromes. Specifically, it focused on cases where FFR was considered unnecessary. Notably, it included patients who underwent diagnostic angiography at a center with a high prevalence of physiology-guided PCI, accounting for approximately 15% of cases. Thus, our cohort represented lesions that were thought not to be ambiguous enough for physiological assessment on the initial angiography.

In our cohort a certain rate of functional misclassification was observed: Of the 488 lesions assessed, 90% had a definitive revascularization decision corroborating the true functional status. Meanwhile, 5% of cases with functionally relevant stenoses were left untreated, while in 5% of cases unnecessary revascularization was indicated. Similar rates of inaccuracies were observed regardless of the different arbitrarily selected “optimal” angiographic cut-off values.

One of the main issues of relying solely on angiography is the assumption that a decision based on the two-dimensional luminogram of the epicardial coronary artery can accurately capture the complexity of myocardial perfusion system. This includes not only the evaluation of epicardial vessels but also the microvascular compartment and the assessment of the amount of viable myocardium present.

The traditional definition of significant coronary artery obstruction is based on physiological principles derived from animal experiments in the early 1970s (19) and the applicability of these principles to the typical patient cohort undergoing coronary angiography is far from obvious and lacks accuracy (8). Angiogram-based lesion assessment is subject to significant intra- and interobserver variability, which limits its standardization (20).

Additionally, due to the complexity of the coronary circulation and myocardial perfusion, anatomical assessments alone are limited in their ability to accurately determine the global relevance of a lesion, regardless of carefully selected cut-off values or improved accuracy of anatomical measurements (21).

For those patients who do undergo invasive coronary angiography, relying solely on angiogram-based decisions is no longer sufficient in the modern era of CCS management: functional assessment of both the macrovasculature and microvasculature plays an integral role in determining the correlation between symptoms clinical status and angiographic findings. In turn, this facilitates a more integrated understanding of the patient's vascular health and shaping effective, personalized treatment strategies.

This study has some limitations that need to be acknowledged. Firstly, our aim of enrolling consecutive patients meant that not all cases were suitable for three-vessel angiogram QFR analysis, with three-vessel QFR being available in 160 patients, representing 84%. Specifically, some angiograms lacked the necessary projections and angulation between acquisitions, as well as exhibiting obscured segments of the coronary vessel acquisitions due to overlap. These factors are crucial for accurate QFR analysis, and their absence was a key reason for the exclusion of certain vessels from the study. Secondly, in some cases angiograms were performed without administration of intracoronary nitro-glycerine, which might have an impact on the accuracy of the QFR measurement. Thirdly, detailed characterization of coronary artery disease, i.e., diffuse disease, calcification, tandem stenoses, etc., which could give deeper understanding of potential causes of discrepancies, is not available. Finally, clinical follow-up of patients is not available, therefore the clinical impact of under- or overtreatment has not been evaluated, however it can be speculated based on previous literature, where large studies have demonstrated negative impact of functional over- and undertreatment on longterm clinical outcomes.

Despite these limitations, the findings of this study highlight the potential errors that can result from relying solely on angiographic findings in clinical decision making. This is true even when operators, with extensive experience in intravascular physiology as was the case in the study center. Accordingly, our study highlights the potential value of incorporating default functional guidance as an effective approach to provide optimal diagnostic and treatment strategies in patients with CSS.

## Conclusions

In a cardiac center with a high utilization rate of intravascular diagnostic modalities, 10% of revascularization strategies were deemed functionally inappropriate in cases where such modalities were not employed due to operator's discretion. This mismatch was most prevalent in the LAD territory. These findings underscore the potential benefits of routinely incorporating angiography-based functional assessments into the management of revascularization decisions in Chronic Coronary Syndrome.

## Data Availability

The datasets presented in this article are not readily available because The data supporting this article may be shared upon reasonable request to the corresponding author, subject to the necessary ethical approvals. Requests to access the datasets should be directed to gabor.g.toth@medunigraz.at.
